# The Dependence of NiMo/Cu Catalyst Composition on Its Catalytic Activity in Sodium Borohydride Hydrolysis Reactions

**DOI:** 10.3390/ma17174353

**Published:** 2024-09-03

**Authors:** Zita Sukackienė, Gitana Valeckytė, Virginija Kepenienė, Irena Stalnionienė, Vitalija Jasulaitiene, Jūratė Vaičiūnienė, Loreta Tamašauskaitė-Tamašiūnaitė, Vidas Pakštas, Eugenijus Norkus

**Affiliations:** Center for Physical Sciences and Technology, Saulėtekio Ave. 3, LT-10257 Vilnius, Lithuania; zita.sukackiene@ftmc.lt (Z.S.); gitana.valeckyte@ftmc.lt (G.V.); vitalija.jasulaitiene@ftmc.lt (V.J.); loreta.tamasauskaite@ftmc.lt (L.T.-T.);

**Keywords:** electroless plating, non-noble metals, catalysis, H_2_ production, boron-hydride

## Abstract

The production of high-purity hydrogen from hydrogen storage materials with further direct use of generated hydrogen in fuel cells is still a relevant research field. For this purpose, nickel-molybdenum-plated copper catalysts (NiMo/Cu), comprising between 1 and 20 wt.% molybdenum, as catalytic materials for hydrogen generation, were prepared using a low-cost, straightforward electroless metal deposition method by using citrate plating baths containing Ni^2+^–Mo^6+^ ions as a metal source and morpholine borane as a reducing agent. The catalytic activity of the prepared NiMo/Cu catalysts toward alkaline sodium borohydride (NaBH_4_) hydrolysis increased with the increase in the content of molybdenum present in the catalysts. The hydrogen generation rate of 6.48 L min^−1^ g_cat_^−1^ was achieved by employing NiMo/Cu comprising 20 wt.% at a temperature of 343 K and a calculated activation energy of 60.49 kJ mol^−1^ with remarkable stability, retaining 94% of its initial catalytic activity for NaBH_4_ hydrolysis following the completion of the fifth cycle. The synergetic effect between nickel and molybdenum, in addition to the formation of solid-state solutions between metals, promoted the hydrogen generation reaction.

## 1. Introduction

Hydrogen (H_2_) became the first potential alternative to fossil fuels and has emerged as a promising and pivotal alternative energy carrier [1]. The primary application of hydrogen as a fuel was started in the aerospace industry, with subsequent research and experimentation conducted in vehicles. In addition, extensive research was conducted on the use of hydrogen as a fuel in fuel cells [2]. Nowadays, the global demand for hydrogen reaches 95 Mt per year (2022), with a nearly 3% increase year on year [3]. However, the majority of hydrogen is produced from natural gas (62%), coal (19%), or naphtha reforming (18%). Nevertheless, these methods inevitably lead to the emission of CO_2_ and thus to the greenhouse effect, which is why research into new, alternative approaches to the production of low-emission hydrogen is relevant today [4,5,6]. Since H_2_ does not exist in its molecular form in nature, consequently, a variety of techniques, including electrochemical, thermochemical, photochemical, photocatalytic, and photoelectrochemical processes, have been subjected to extensive investigation and improvement on a global scale with regard to their potential for hydrogen production [7]. Furthermore, hydrogen fuel cells have already been implemented in various sectors, including aviation, electrical power generation, and automotive technology [1,7,8]. However, hydrogen is frequently employed as a compressed gas, which presents a number of challenges, including purification, transportation, and storage. Therefore, research has been focused on the development of solid-state hydrogen storage materials and direct hydrogen generation reactions [9,10]. The environmentally friendly chemical boron hydrides (NaBH_4_ (10.8 wt.%), LiBH_4_ (18 wt.%), NH_3_-BH_3_ (19.6 wt.%), etc.) have been considered promising for hydrogen storage and use in portable fuel cells due to their high H_2_ capacity [11,12]. Sodium borohydride (NaBH_4_) has been identified as a highly suitable material for hydrogen storage due to a number of favorable characteristics, including its high hydrogen content, cost-effectiveness, environmental compatibility, stability in alkaline solutions, non-flammability, and renewability [13,14,15,16,17,18,19,20,21,22,23,24,25,26,27,28,29,30,31,32,33,34,35,36,37,38,39,40,41]. The production of hydrogen from NaBH_4_ hydrolysis is described by the following reaction (1), as outlined in reference [9,21,25,26,27,28,29,30]:

NaBH_4_ + 2H_2_O → 4H_2_ + NaBO_2_, ΔH° = −216.7 kJ mol^−1^ (1)

The efficiency of the borohydride hydrolysis reaction is contingent upon two primary factors: the activation energy of the reaction and the quantity of hydrogen released. In contact with water, the NaBH_4_ starts producing H_2_; however, the H_2_ gas rate is very low and the water-NaBH_4_ solution is unstable. Raising the pH by adding NaOH stabilizes the NaBH_4_ solution. Therefore, the pivotal factor for the production of highly purified hydrogen through NaBH_4_ hydrolysis is the discovery of selective catalysts that could accelerate the generation of H_2_ in alkaline solution [42]. The development of efficient catalysts plays a pivotal role in the conversion and utilization of clean energy [10]. A number of noble metals, including Pt, Ru, Pd, and Rh, have been identified as highly effective catalysts for accelerating the hydrolysis of NaBH_4_ due to their favorable performance, stability, and tolerance to deactivation [15,17,19,20,26]. Furthermore, the dispersion of nano-sized noble metals on carbon results in the catalyst having an enhanced electroactive surface area, which in turn exhibits an improved hydrogen generation rate [43,44,45]. However, the high cost, low abundance, and rapid surface poisoning of these metals have prompted the development of new non-noble catalysts with activity comparable to that of noble metals [40,46,47,48,49]. Among the metals under consideration, those belonging to the transition metal family, including Ni, Co, Fe, Mn, Cr, or Mo, have attracted the attention of researchers [9,13,18,21,22,23,24,31,32,33,34,35,36,37,39,41,47,50,51]. In this group of non-noble metal catalysts, nickel has been selected due to its advantageous characteristics, including catalytic activity, ferromagnetic properties, and reduced operating costs in recycling [52,53]. Moreover, alloyed transition metals create a synergistic effect to enhance catalytic activity [54,55]. Ni–Mo composites show higher electrocatalytic activity than pure Ni due to the improved intrinsic activity of the material. This is explained by the change in electron density in the d-shell when Ni is alloyed with Mo [54,55]. A variety of techniques have been documented in the scientific literature for the production of pure metal films or a few component coatings of these metals in combination with one another. A. Didehban et al. successfully synthesized bimetallic NiCo on magnetic supports using a heterogeneous deposition-precipitation (HDP) method. Furthermore, the activation energy of the sodium borohydride hydrolysis reaction was established to be 63.27 kJ mol^−1^ when employing the Co-Ni/MSAC catalyst, as reported in reference [32]. Z. Liang et al. proposed the NiB/NiFe_2_O_4_ catalyst, prepared via the impregnation chemical reduction method [37]. The NiB/NiFe_2_O_4_ catalyst exhibited an activation energy of 72.52 kJ mol^−1^ for the sodium borohydride hydrolysis reaction. H. Guo et al. presented a method for the preparation of Ag-Ni core and shell nanoparticles, which involved the thermal decomposition of nickel and silver salts using a one-pot seed growth approach. The activation energy for hydrolysis of NaBH_4_ catalyzed by this substance was determined to be 57.62 kJ mol^−1^ [38]. The authors posit that the increased catalytic activity of the combined materials can be attributed to the synergistic properties that are induced by the intimate contact and interaction between the different components [32,37,38].

This paper presents an investigation of the catalytic activity of Ni-Mo catalysts towards the hydrolysis reaction of NaBH_4_ to produce hydrogen fuel. Non-noble catalysts were constructed by selecting the combination of Ni and Mo coatings on the Cu substrate, which was chosen as a substrate because it provides a stable base. In addition, it is easy to deposit the identical Ni coatings on the Cu surface. Molybdenum was chosen to increase the efficiency of the NaBH_4_ hydrolysis reaction. It should be noted that the Ni sublayer is also required to deposit molybdenum. The NiMo(1)/Cu, NiMo(5)/Cu, NiMo(10)/Cu, NiMo(15)/Cu, and NiMo(20)/Cu catalysts were prepared with varying Mo content (1, 5, 10, 15, and 20 wt.%, respectively) by a simple and cost-effective approach—electroless metal plating using morpholine borane as a reducing agent. The catalytic activity of the NiMo/Cu catalysts was examined in the generation of hydrogen from alkaline NaBH_4_ solutions during hydrolysis at varying temperatures. Furthermore, the structure of the prepared catalysts was characterized using a range of analytical techniques, including inductively coupled plasma optical emission spectroscopy (ICP-OES), field emission scanning electron microscopy (FESEM), X-ray photoelectron spectroscopy (XPS), and X-ray diffraction (XRD).

## 2. Materials and Methods

### 2.1. Chemicals

Copper sheet (Cu, 99.8% purity) and sodium molybdate dihydrate (Na_2_MoO_4_∙2H_2_O, 99.5%) were purchased from Sigma-Aldrich (Saint Luise, MO, USA). Sulphuric acid (H_2_SO_4_, 96%), hydrochloric acid (HCl, 35–38%), nickel sulfate hexahydrate (NiSO_4_∙6H_2_O, 98%), morpholine borane (C_4_H_8_ONH∙BH_3_, 97%), palladium chloride (PdCl_2_, 99.95%), sodium citrate (Na_3_C_6_H_5_O_7_, 99%), and sodium hydroxide (NaOH, 98.8%) were purchased from Chempur Company (Karlsruhe, Germany). All chemicals were of analytical grade and were used directly without further purification.

### 2.2. Fabrication of Catalysts

**Scheme 1:**
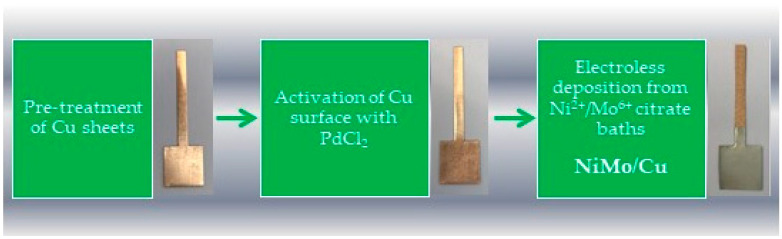
The electroless plating process of NiMo coatings on the Cu sheets.

Scheme 1 shows the electroless plating process of NiMo coatings on the Cu sheets using morpholine borane as a reducing agent in citrate solutions.

Briefly, the copper sheets intended for Ni-Mo deposition were prepared in the following manner: initially, Cu sheets in size of 1 × 1 cm were treated with a calcium magnesium oxide comprising between 50% and 100% of the material, otherwise known as “Vienna Lime” (Kremer Pigments GmbH & Co. KG, Aichstetten, Germany). They were then rinsed with deionized water and kept in a 10% HCl solution at room temperature for one minute, during which time any inorganic impurities were removed. They were subsequently washed and dried. Subsequently, the pre-treated Cu sheet was activated with Pd(II) ions through immersion in a 0.5 g L^−1^ PdCl_2_ solution for a period of 5 s. Following this, the sheet was rinsed with deionized water and placed in an electroless plating solution. The electroless deposition of Ni-Mo was conducted on Cu sheets using the plating solutions outlined in Table 1, with morpholine borane (MB) employed as the reducing agent.

It can be observed that the composition of the plating solution remained unvaried, with the exception of the concentration of Na_2_MoO_4_·2H_2_O. As a consequence, catalysts with a Mo content of 1−20 wt.% were prepared, comprising the following compositions: NiMo(1)/Cu, NiMo(5)/Cu, NiMo(10)/Cu, NiMo(15)/Cu, and NiMo(20)/Cu. In all cases, the pH of the plating solutions was 7 (as determined at room temperature), the temperature of the deposition bath was 50 °C, and the deposition time was 12 min for all catalysts under investigation.

### 2.3. Characterization of Catalysts

The morphology of the prepared NiMo/Cu catalysts was investigated via scanning electron microscopy (SEM) using an SEM workstation SEM TM 4000 Plus (HITACHI) with an energy dispersive X-ray (EDX) spectrometer. The metal loadings were determined via inductively coupled plasma optical emission spectroscopy (ICP-OES) analysis. The ICP-OES spectra were recorded using an Optima 7000DV spectrometer (Perkin Elmer, Waltham, MA, USA) at wavelengths of λ_Ni_—231.604 nm and λ_Mo_—202.031 nm.

X-ray photoelectron spectroscopy (XPS) was employed to characterize a series of NiMo catalysts, utilizing a Kratos AXIS Supra+ spectrometer (Kratos Analytical, Manchester, UK, 219) with monochromatic Al Kα (1486.6 eV) X-ray radiation, powered at 225 W. The base pressure in the analysis chamber was less than 1 × 10^−9^ mbar and a low electron flood gun was used as a charge neutralizer. The survey spectra for each sample were recorded at a pass energy of 160 eV with a 1 eV energy step and high-resolution spectra (pass energy—20 eV, in 0.1 eV steps) over individual element peaks. The binding energy scale was calibrated by setting the adventitious carbon peak at 284.8 eV. The XPS data were converted to VAMAS format and processed using Avantage software version 5.9922 (Thermo Scientific, East Grinstead, UK).

The XRD patterns of the studied catalysts were obtained using an X-ray diffractometer Smart-Lab (Rigaku, Japan, 2011) equipped with an X-ray tube with a 9 kW rotating Cu anode. The measurements were conducted using the Bragg–Brentano geometry with a graphite monochromator on the diffracted beam and a step scan mode with a step size of 0.02 (in 2-theta scale) and a counting time of 1 s per step. The measurements were conducted in the 2-theta range of 10–75°. Phase identification was performed using the software package PDXL version 1.8.0.3 (Rigaku, Japan) and the ICDD powder diffraction database PDF4+ (2023 release).

### 2.4. Hydrolysis Measurements of NaBH_4_

A thermostatically controlled, hermetically sealed flask with an outlet linked to the MilliGascounter was employed to collect the produced hydrogen gas from a solution comprising 15 mL of 5 wt.% NaBH_4_ + 0.4 wt.% NaOH. The volume of the released hydrogen catalyzed by the prepared NiMo/Cu catalysts was measured using a MilliGascounter (Type MGC-1 V3.2 PMMA, Ritter, Bochum, Germany) connected to a personal computer. For all measurements, the prepared catalysts were placed in an alkaline sodium borohydride solution of the designated temperature and stirred with a magnetic stirrer. The hydrogen generation rate (HGR) was measured at a working solution within the temperature range of 303 to 343 K in order to determine the activation energy of the reaction. The activation energy (*E*_a_) is defined as the minimum energy content required for a reacting species to carry out a given reaction. In general, higher reaction activity is associated with lower “*E*_a_” values. The activation energy of the hydrolysis reaction of NaBH_4_ was calculated by using the Arrhenius equation based on the ln(k) versus 1/T dependence, as outlined in Equation (2):

ln k = ln A − *E*_a_/(RT) (2)

where A—the pre-exponential factor, *E*_a_—the activation energy (J), R—the general gas constant (8.314 J mol^−1^ K^−1^), and k—the reaction coefficient.

## 3. Results and Discussion

### 3.1. Coatings, Microstructure and Morphology Studies

The primary objective of this study was to develop an effective catalyst for the generation of hydrogen from an alkaline sodium borohydride solution using a straightforward methodology. Consequently, two-component NiMo coatings with varying Mo concentrations were deposited on Cu sheets via a straightforward electroless deposition process, employing morpholine borane as the reducing agent in a citrate solution. Table 2 presents the elemental composition of the prepared NiMo(1)/Cu, NiMo(5)/Cu, NiMo(10)/Cu, NiMo(15)/Cu and NiMo(20)/Cu catalysts. The molybdenum content was varied within the range of 1 to 20 wt.% molybdenum. Accordingly, the amount of nickel in the coatings was in the range of 99 wt.% to 80 wt.%. The deposition time—12 min—was observed as the optimal cause. It is important to highlight that upon further augmentation of the molybdenum precursor concentration in the plating bath, the reaction does not start to occur. It could be caused by the Mo and its compounds adsorbing on the catalytically active surface and preventing chemical precipitation. Molybdenum itself is not catalytically active in the morpholine borane oxidation reaction [10,11,12,13,14]. Consequently, it was not feasible to deposit coatings with a greater amount of molybdenum than 20 wt.%. Moreover, higher Mo content in Ni–Mo composites could deteriorate the corrosion resistance due to surface roughness and grain refinement [56]. It was observed that the deposition rate decreased from 3.65 to 3.1 µm h^−1^ during the preparation of catalysts containing 1 and 5 wt.% Mo, respectively. Additionally, the total content of Ni and Mo decreased from 652.03 to 555.55 µg cm^−2^ depending on the content of Mo. It is known that the oxidation of morpholine by borane is catalyzed by nickel. However, when nickel is co-located with molybdenum, the catalytically active Ni centers are covered by Mo, resulting in a decrease in the reaction rate. Conversely, when the molybdenum content in the coating reached 10−20 wt.%, the deposition rate remained constant at approximately 2.75 µm h^−1^. Moreover, the general Ni and Mo contents in the coatings exhibit minimal variation, remaining within the range of 473.31 to 490.35 µg cm^−2^ (Table 2). The findings confirm that an increase in the molybdenum content of the catalyst results in a decline in the electroless deposition rate, which ultimately leads to the cessation of the reaction at higher concentrations of Mo^6+^. However, nickel–molybdenum alloys have been demonstrated to be effective catalysts for the hydrolysis of borohydrides.

**Figure 1:**
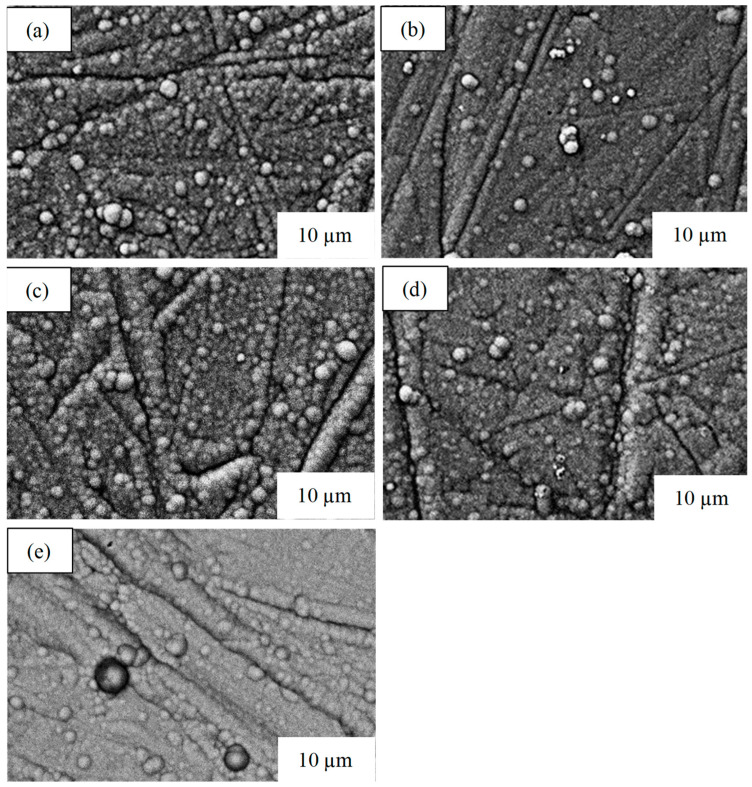
SEM images of prepared (**a**) NiMo(1), (**b**) NiMo(5), (**c**) NiMo(10), (**d**) NiMo(15), and (**e**) NiMo(20) catalysts.

The morphology and particle size of the prepared NiMo/Cu catalysts were assessed through the utilization of scanning electron microscopy (SEM) images. Figure 1 shows the SEM images of the prepared NiMo(1)/Cu, NiMo(5)/Cu, NiMo(10)/Cu, NiMo(15)/Cu, and NiMo(20)/Cu catalysts. As illustrated in Figure 1, the surface morphology of the prepared catalysts is observed to be compact, smooth, and free of cracks, exhibiting distinctive multi-layered cauliflower-like structures. Furthermore, the SEM images demonstrate that all of the prepared coatings are composed of particles of varying dimensions that coalesce to form oval agglomerates (Figure 1a–e). It is believed that an increased number of agglomerates on the surface will result in an enhanced active surface area, thereby improving the efficacy of the catalysts. The estimated average size range of the agglomerates on the surface is 0.286 to 2.143 μm.

### 3.2. XPS Analysis of the Prepared Catalysts

**Figure 2:**
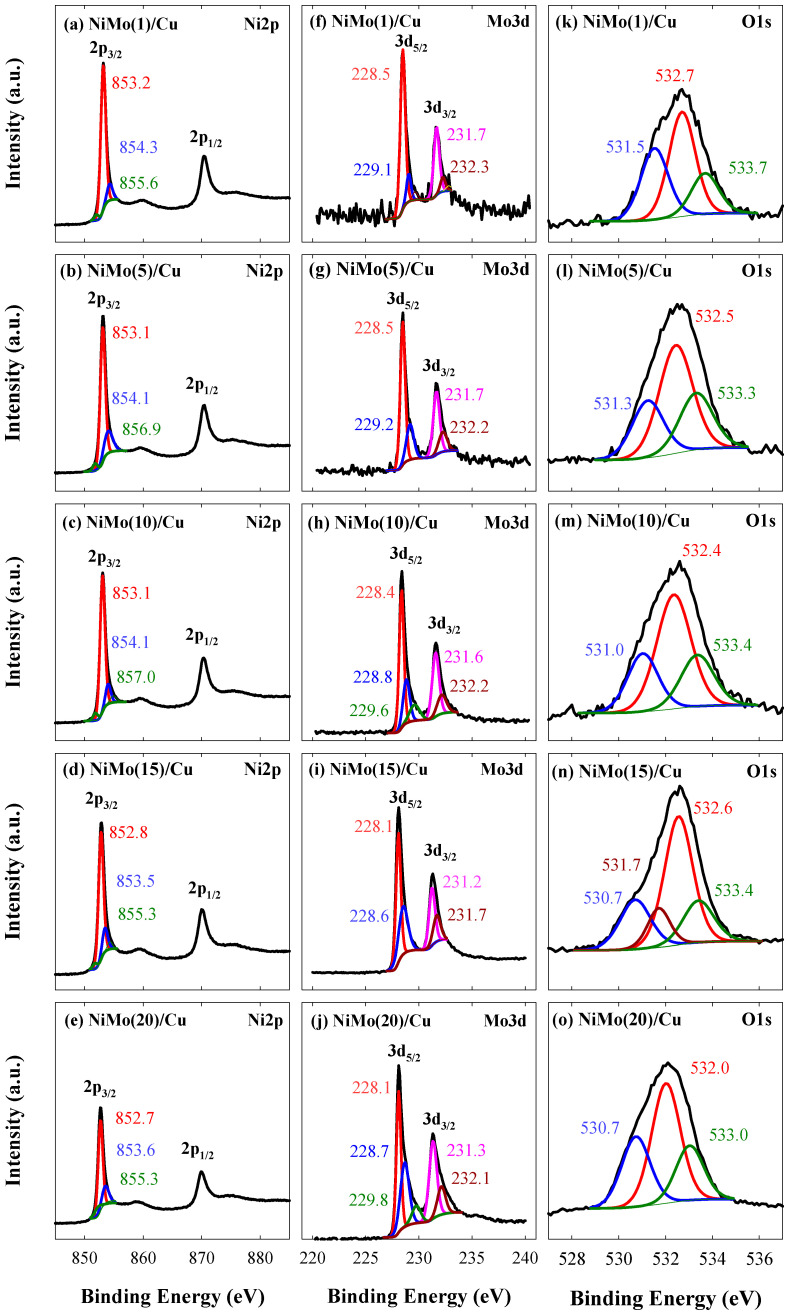
High-resolution XPS spectra of Ni 2p (**a**–**e**), Mo 3d (**f**–**j**), and O 1s (**k**–**o**) for different NiMo/Cu catalysts.

The electronic state and surface interaction between the atoms in coatings were investigated via XPS spectra analysis in order to gain insight into the promoting effect of Mo on the catalytic activity. Figure 2 reports the X-ray photoelectron spectra of the Ni 2p (a–e), Mo 3d (f–j), and O 1s (k–o) electron levels in the NiMo(1)/Cu), NiMo(5)/Cu, NiMo(10)/Cu, NiMo(15)/Cu, and NiMo(20)/Cu catalysts. As can be observed in Figure 2a–e, the two intense peaks in the Ni 2p spectra at binding energies of 852.7 ± 0.5 eV and 870.0 ± 0.5 eV with a split spin-orbit component of Δ_metal_ = 17.3 eV were assigned to Ni^0^ 2p_3/2_ and Ni^0^ 2p_1/2_, respectively [57,58]. The lower intensity Ni 2p_3/2_ binding energy peaks originating at 853.2–855.5 eV were assigned to Ni^2+^, as reported in previous studies [57] (Figure 2a–c).

In the case of Mo 3d XPS data, the doublet peaks originating at 228.1 ± 0.4 eV and 231.3 ± 0.4 eV for Mo 3d_5/2_ and Mo 3d_3/2_ transitions, respectively, demonstrated the presence of Mo^0^ metallic phase in all samples [58]. The binding energy peaks at 229.2 ± 0.6 and 232.1 ± 0.4 eV for Mo 3d_5/2_ and Mo 3d_3/2_ transitions, respectively, may be assumed to be Mo^4+^ and indicated that apart from Mo^0^, Mo in the NiMo coatings existed as MoO_2_ [58]. Moreover, in all cases, the spin-orbital splitting transition peaks in the O 1s XPS spectra, with the binding energy peaks at 530.7–531.7 eV, correspond to the lattice oxygen species, while the dominant peaks at 532.0 ± 0.5 eV may be assumed to be hydroxides and defect oxides [57,58,59]. Higher binding energy peaks located at approximately 533.0 ± 0.7 eV are usually attributed to the presence of the surface of adsorbed O_2_, H_2_O, and CO_2_ (Figure 2) [59].

### 3.3. XRD Analysis of the Prepared Catalysts

**Figure 3:**
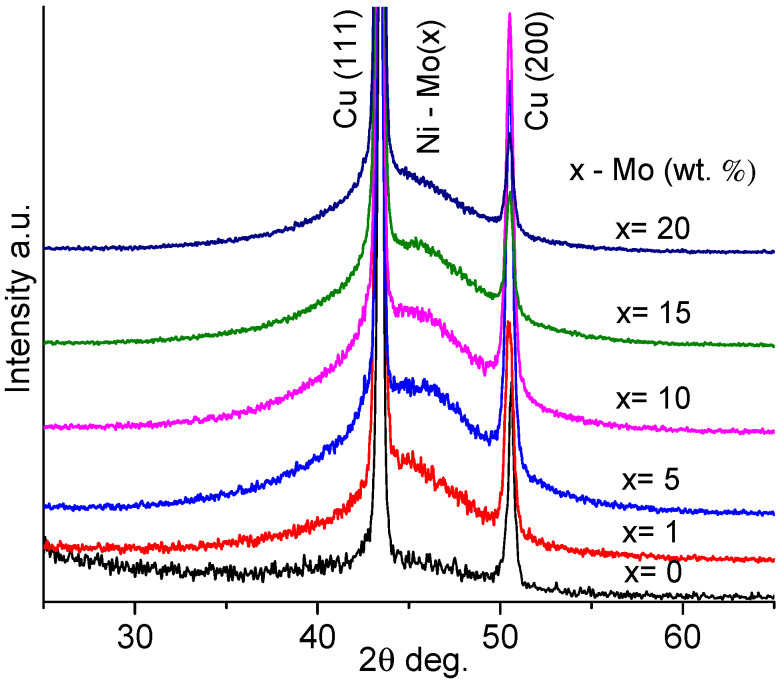
XRD patterns of the prepared Ni/Cu and NiMo/Cu catalysts.

It should be noted that the XRD analysis revealed highly comparable crystallographic patterns across all of the prepared catalysts (Figure 3). As can be seen, two intense diffraction peaks at 2-theta = 43.4 and 50.5° are observed in all profiles and are assigned to the (111) and (200) crystallographic planes of crystalline Cu from the Cu sheet (JCPDS No. 04-0836) [60]. No distinct sharp peak is observed for the Ni and Mo, indicating a low level of crystallinity where the size of the crystalites does not exceed 5 nm. As the concentration of Mo in the catalyst rises, the nickel lattice may undergo tensile strain, and the Ni lattice may deform. However, the XRD patterns of NiMo/Cu catalysts show a broad and diffuse peak centered at about 44–51°; therefore the lattice parameters of this compound cannot be accurately estimated. The obtained broad and diffuse Ni-Mo(x) peak could be attributed to the amorphous compounds of Ni and Mo or to the nickel-molybdenum solid-state liquid formation [55,61,62].

### 3.4. Catalysts Activity toward NaBH_4_ Hydrolysis

**Figure 4:**
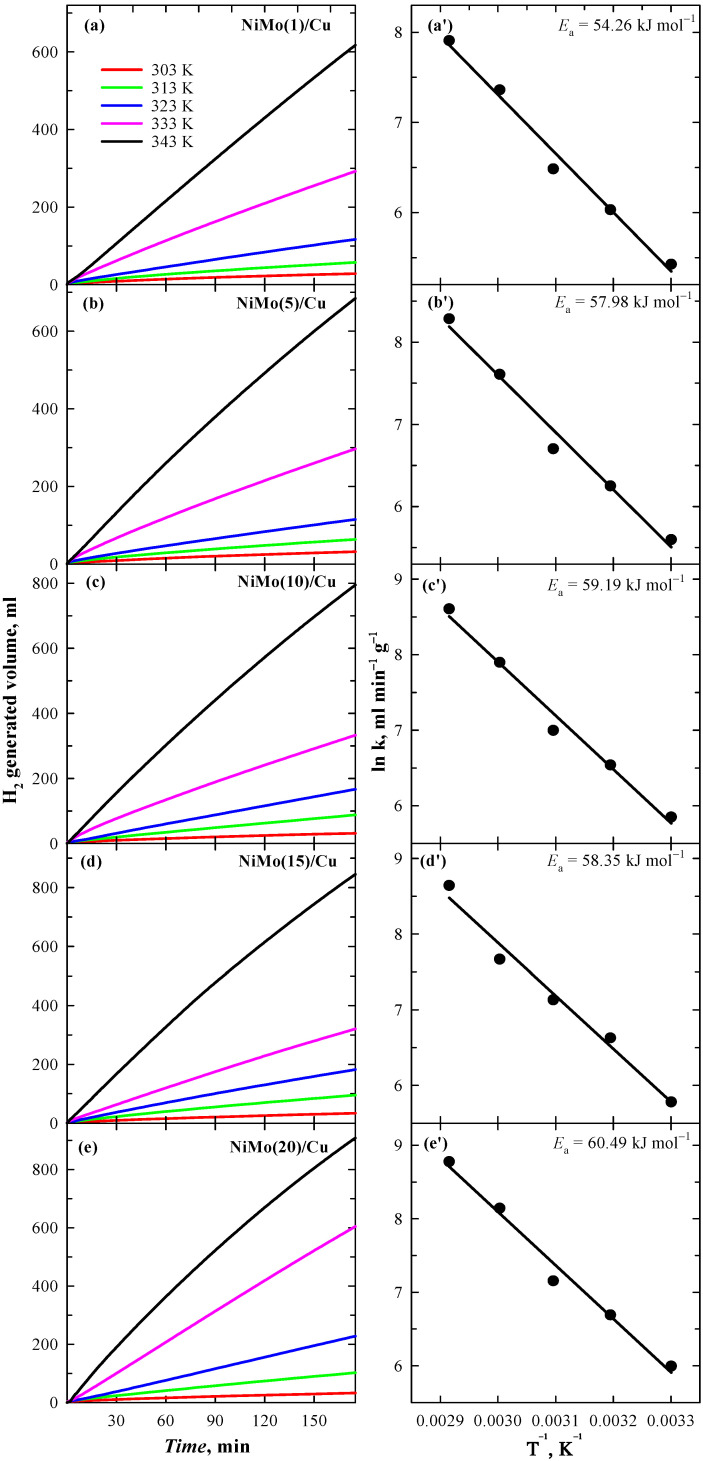
H_2_ generation from 15 mL 5 wt.% NaBH_4_ + 0.4 wt.% NaOH catalyzed by the NiMo(1)/Cu (**a**), NiMo(5)/Cu (**b**), NiMo(10)/Cu (**c**), NiMo(15)/Cu (**d**), and NiMo(20)/Cu (**e**) catalysts at different temperatures. (**a′**–**e′**) The corresponding Arrhenius plots.

The catalytic performances of the prepared NiMo/Cu catalysts toward NaBH_4_ hydrolysis were evaluated in the solution containing 5 wt.% NaBH_4_ + 0.4 wt.% NaOH in the temperature range of 303–343 K. Figure 4 shows the temperature-time dependent volume of H_2_ generated (mL) from the NaBH_4_ solution obtained on the NiMo(1)/Cu (a), NiMo(5)/Cu (b), NiMo(10)/Cu (c), NiMo(15)/Cu (d). and NiMo(20)/Cu (e) catalysts at different temperatures. As can be seen from this figure, the hydrogen generation rate (HGR) increases in all cases as the temperature increases from 303 to 343 K. The summarized data for the HGR are given in Table 3. The data show that all the catalysts studied exhibited catalytic performance toward the hydrolysis of NaBH_4_. However, when the catalysts are compared, the results clearly show that the catalyst with the highest content of molybdenum exhibits the most effective catalytic activity toward the NaBH_4_ hydrolysis reaction, ranging from 0.4 to 6.48 L min^−1^ g_cat_^−1^ at the temperature of 303–343 K, respectively. The results showed that increasing the temperature of the operating bath from 303 to 343 K resulted in a 22-fold increase in the rate of hydrogen generation. Figure 4a′–e′ shows the Arrhenius plots for all of the catalysts investigated. The rate of hydrogen gas released during the reaction (k) was calculated using the known amount of hydrogen produced after 30 min at each temperature. The activation energy of the electrochemical process (*E*_a_, kJ mol^−1^) was then calculated using the Arrhenius Equation (2). It was observed that the activation energy of the reaction increased with increasing Mo content in the catalyst (Table 3). The lowest *E*_a_ value of 54.26 kJ mol^−1^ was obtained for the NiMo(1)/Cu catalyst (Figure 4a′); however, the lowest hydrogen generation rate of 0.23–2.72 L min^−1^ g_cat_^−1^ was also observed for this catalyst in the temperature range of 303–343 K (Table 3). Although the NiMo(1)/Cu catalyst required the least amount of energy to initiate the reaction, the highest amount of released hydrogen was obtained with the NiMo(20)/Cu catalyst, although its activation energy was 60.49 kJ mol^−1^. As the temperature was increased from 303 to 343 K, the HGR for the NiMo(20)/Cu catalyst was calculated to range from 0.40 to 6.48 L min^−1^ g_cat_^−1^. In general, all of the investigated catalysts, NiMo(1)/Cu, NiMo(5)/Cu, NiMo(10)/Cu, NiMo(15)/Cu, and NiMo(20)/Cu, exhibited catalytic activity for sodium borohydride hydrolysis; however, the NiMo(20)/Cu catalyst demonstrated 19%, 13%, 33%, and 43%, higher catalytic efficiency for hydrogen generation than NiMo(15)/Cu, NiMo(10)/Cu, NiMo(5)/Cu, and NiMo(1)/Cu, respectively, at the lowest temperature (303 K) of the working bath.

When the working bath was maintained at 343 K, the NiMo(20)/Cu catalyst exhibited markedly enhanced catalytic efficiencies for hydrogen generation, with values 13%, 16%, 39%, and 58% higher than those observed for the NiMo(15)/Cu, NiMo(10)/Cu, NiMo(5)/Cu, and NiMo(1)/Cu catalysts, respectively. It is noteworthy that the higher molybdenum content of approximately 10–20 wt.% in the NiMo/Cu catalysts results in a higher hydrogen generation rate. The data obtained demonstrate that the successful interstitial insertion of larger amounts of Mo into nickel coatings resulted in a notable enhancement in catalytic activity toward H_2_ generation. It has been demonstrated that a low activation energy does not necessarily result in a high value of released hydrogen. This phenomenon may be attributed to the enhanced synergistic effect of the metals present in the catalysts. It has been observed that transition-metal-based binary alloys exhibit notable catalytic activity, which is primarily attributed to their distinctive characteristics, including a high concentration of unsaturated sites and an amorphous structure with short-range order, long-range disorder, and chemical stability [33,41,63]. The activation energies of the NiMo/Cu catalysts are among the most active compounds recently reported for non-noble metal electrocatalysts for borohydride hydrolysis, as shown in Table 4.

The *E*_a_ values obtained for all of the investigated catalysts in the range of 54.26 to 60.49 kJ mol^−1^ are comparable to those reported for other materials, including CoMoB/C [39], Ni–Ru/50WX8 [38], Ni–Fe–B [33], and Ag-Ni core-shell [40]. The values obtained for the investigated catalysts are higher than those for materials such as Co-Ni/MSAC (63.27 kJ mol^−1^) [32], CoFeMo/Ni (66.80 kJ mol^−1^) [41], bulk Ni catalysts (67.90 kJ mol^−1^ and 72.52 kJ mol^−1^) [27,31], nickel-cobalt (alloys) (68.84 kJ mol^−1^) [50], or CoFeMo/Ni (66.8 kJ mol^−1^) [41]. Nevertheless, noble metals such as Pt, Ru, and Ag have been demonstrated to reduce the activation energy of the borohydride hydrolysis reaction (see Table 4, [38,40]). However, we present a non-noble, inexpensive NiMo composite with a comparable efficiency for hydrogen generation from an alkaline sodium borohydride solution.

**Figure 5:**
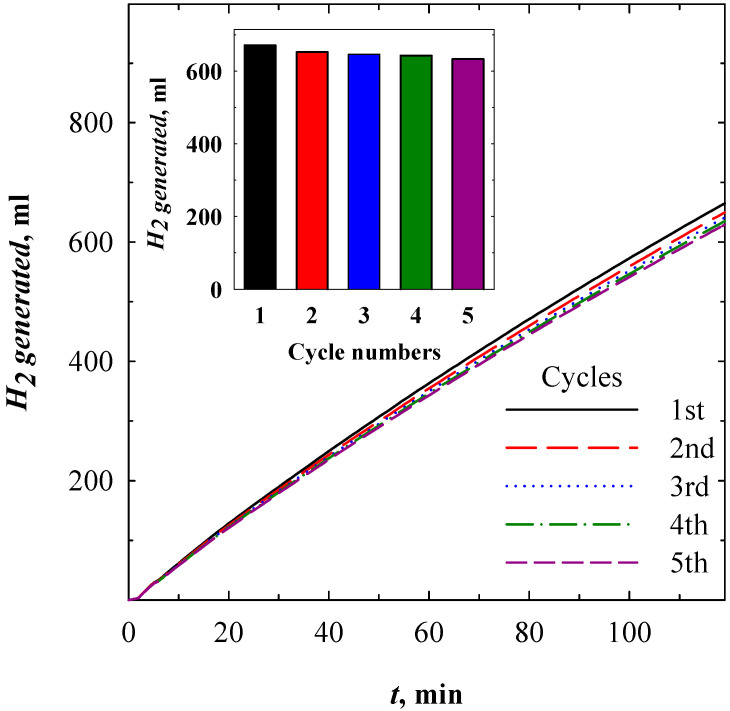
Reusability of the NiMo(20)/Cu catalyst after five cycles in 5 wt.% NaBH_4_ + 0.4 wt.% NaOH solution.

The investigation revealed that the NiMo(20)/Cu catalyst exhibited the highest efficiency in catalyzing the NaBH_4_ hydrolysis reaction, resulting in the highest H_2_ generation volume. Consequently, a stability and reusability test was conducted on the NiMo(20)/Cu catalyst, given its pivotal role in the practical application of the hydrogen generation system. A stability test was conducted by reusing the NiMo(20)/Cu catalyst in five cycles of hydrogen generation in a 5 wt.% NaBH_4_ and 5 wt.% NaOH solution at 343 K. At the end of each cycle, the catalyst was washed several times with deionized water to remove any deposited sodium metaborate, which could potentially lead to clogging of the active centers, and then dried and reused. Figure 5 illustrates that the catalytic activity of the NiMo(20)/Cu catalyst remains largely unaltered.

The NiMo(20)/Cu catalyst exhibited a remarkable retention of 94% of its initial catalytic activity for NaBH_4_ hydrolysis at the end of the fifth cycle. The slight decline in activity may be attributed to the precipitation of a minute quantity of sodium metaborate byproduct on the catalyst surface. This phenomenon results in a reduction in the number of active centers, which ultimately leads to a decline in the catalyst’s activity. Another significant factor contributing to the observed decline in catalyst activity is the surface oxidation of nickel-based catalysts, which results in deactivation. Therefore, the loss of catalytic activity centers, surface oxidation, or the loss of catalytic NiMo(20)/Cu nanoparticles during separation and post-run washing also contributed to a reduction in the hydrogen generation rate.

In conclusion, the favorable activation energies obtained in the present work on the proposed NiMo(1)/Cu, NiMo(5)/Cu, NiMo(10)/Cu, NiMo(15)/Cu, and NiMo(20)/Cu catalysts, which lead to an improvement in catalytic hydrolysis performance, can be attributed to the distinctive characteristics of the surface and the synergistic effects of Ni and Mo.

## 4. Conclusions

In this study, we successfully prepared NiMo/Cu catalysts with varied molybdenum content, ranging from 1 to 20 wt.%, using the low-cost, simple electroless metal plating method. This method employs morpholine borane as a reducing agent in citrate solutions. The principal findings are as follows:

It was found that the maximum amount of molybdenum, 20%, can be reached with further passivation of the catalytic surface. All of the investigated coatings were observed to be compact, devoid of any cracks or defects, and exhibited catalytic activity for the sodium hydrolysis reaction.

The hydrogen generation rate was found to be dependent on the molybdenum content in the catalysts and the temperature of the working solution. Hydrogen generation rates of between 2.72 and 6.48 L min^−1^ g_cat_^−1^ were achieved at a temperature of 343 K by employing the NiMo/Cu catalysts with varying molybdenum content, from 1 to 20 wt.%, respectively.

The activation energy of the borohydride hydrolysis reaction exhibited a slight increase, from 54.26 to 60.49 kJ mol^−1^, as the molybdenum content in the NiMo/Cu catalysts was increased from 1 to 20 wt%.

The deposition of two-component coatings combining Ni and Mo has been demonstrated to result in enhanced performance, specifically in terms of hydrogen generation rate and remarkable stability toward catalytic activity for NaBH_4_ hydrolysis. Consequently, these catalysts represent a promising avenue for hydrogen production from alkaline NaBH_4_ solutions.

## Figures and Tables

**Table 1:** Composition of the electroless plating solution and the deposition parameters of the catalysts.

Catalyst	Composition of Plating Solution (mol L^−1^) and Plating Conditions						
	NiSO_4_	Na_3_C_6_H_5_O_7_	MB	Na_2_MoO_4_	pH	T, °C	t, min
NiMo(1)/Cu	0.1	0.04	0.2	0.0001	7	50	12
NiMo(5)/Cu				0.0005			
NiMo(10)/Cu				0.0008			
NiMo(15)/Cu				0.0010			
NiMo(20)/Cu				0.0012			

**Table 2:** The elemental composition and metal loading of the prepared NiMo/Cu coatings analyzed via ICP-OES.

Catalyst	Element, wt.%		Element Loading, µg cm^−2^			Deposition Rate, µm h^−1^
	Ni	Mo	Ni	Mo	Ni + Mo	
NiMo(1)/Cu	99.20	0.85	646.50	5.525	652.03	3.65
NiMo(5)/Cu	94.60	5.41	525.50	30.05	555.55	3.10
NiMo(10)/Cu	89.99	10.01	425.96	47.35	473.31	2.65
NiMo(15)/Cu	85.46	14.54	419.05	71.30	490.35	2.75
NiMo(20)/Cu	80.31	19.69	391.15	95.90	487.05	2.75

**Table 3:** H_2_ generation rates and activation energies of the prepared catalysts.

Catalyst	*E*_a_, kJ mol^−1^	T, K	v, mL min^−1^	v, L min^−1^ g_cat_^−1^
NiMo(1)/Cu	54.26	303 K	0.30	0.23
		313 K	0.54	0.42
		323 K	0.85	0.65
		333 K	2.05	1.57
		343 K	3.55	2.72
NiMo(5)/Cu	57.98	303 K	0.30	0.27
		313 K	0.58	0.52
		323 K	0.90	0.81
		333 K	2.24	2.01
		343 K	4.40	3.96
NiMo(10)/Cu	59.19	303 K	0.33	0.35
		313 K	0.66	0.69
		323 K	1.04	1.09
		333 K	2.55	2.69
		343 K	5.17	5.46
NiMo(15)/Cu	58.35	303 K	0.32	0.32
		313 K	0.74	0.76
		323 K	1.23	1.25
		333 K	2.10	2.14
		343 K	5.56	5.67
NiMo(20)/Cu	60.49	303 K	0.39	0.40
		313 K	0.78	0.81
		323 K	1.25	1.28
		333 K	3.35	3.44
		343 K	6.32	6.48

**Table 4:** Comparison of activation energy (*E*_a_).

Catalyst	Electrolyte	*E*_a_, kJ mol^−1^	Reference
CoMoB/C	5 wt.% NaBH_4_ + 7 wt.% NaOH	51.00	[39]
Ni–Ru/50WX8	5 wt.% NaBH_4_ + 5 wt.% NaOH	52.73	[40]
Ni–Fe–B	5 wt.% NaBH_4_ + 4 wt.% NaOH	57.00	[33]
Ag-Ni core-shell	10 wt.% NaBH_4_ + 10 wt.% NaOH	57.62	[38]
Co-Ni/MSAC	5 wt.% NaBH_4_ + 5 wt.% NaOH	63.27	[32]
CoFeMo/Ni	5 wt.% NaBH_4_ + 0.4 wt.% NaOH	66.80	[41]
Ni	5 wt.% NaBH_4_ + 0.1 M NaOH	67.90	[31]
Ni-Co (Alloy)	5 wt.% NaBH_4_ + 5 wt.% NaOH	68.84	[50]
Ni	5 wt.% NaBH_4_ + 10 wt.% NaOH	72.52	[37]
NiMo(1)/Cu	5 wt.% NaBH_4_ + 0.4 wt.% NaOH	54.26	this study
NiMo(5)/Cu	5 wt.% NaBH_4_ + 0.4 wt.% NaOH	57.98	this study
NiMo(10)/Cu	5 wt.% NaBH_4_ + 0.4 wt.% NaOH	59.19	this study
NiMo(15)/Cu	5 wt.% NaBH_4_ + 0.4 wt.% NaOH	58.35	this study
NiMo(20)/Cu	5 wt.% NaBH_4_ + 0.4 wt.% NaOH	60.49	this study

## Data Availability

The original contributions presented in the study are included in the article, further inquiries can be directed to the corresponding authors.
